# HypMix: Hyperbolic Representation Learning for Graphs with Mixed Hierarchical and Non-hierarchical Structures

**DOI:** 10.1145/3627673.3679940

**Published:** 2024-10-21

**Authors:** Eric W. Lee, Bo Xiong, Carl Yang, Joyce C. Ho

**Affiliations:** Emory University, Atlanta, GA, USA; University of Stuttgart, Stuttgart, Germany; Emory University, Atlanta, GA, USA; Emory University, Atlanta, GA, USA

**Keywords:** Hyperbolic Representation Learning, Hyperbolic Space, Graph Representation Learning, Hierarchical Structure

## Abstract

Heterogeneous networks contain multiple types of nodes and links, with some link types encapsulating hierarchical structure over entities. Hierarchical relationships can codify information such as subcategories or one entity being subsumed by another and are often used for organizing conceptual knowledge into a tree-structured graph. Hyperbolic embedding models learn node representations in a hyperbolic space suitable for preserving the hierarchical structure. Unfortunately, current hyperbolic embedding models only implicitly capture the hierarchical structure, failing to distinguish between node types, and they only assume a single tree. In practice, many networks contain a mixture of hierarchical and non-hierarchical structures, and the hierarchical relations may be represented as multiple trees with complex structures, such as sharing certain entities. In this work, we propose a new hyperbolic representation learning model that can handle complex hierarchical structures and also learn the representation of both hierarchical and non-hierarchic structures. We evaluate our model on several datasets, including identifying relevant articles for a systematic review, which is an essential tool for evidence-driven medicine and node classification.

## 1 Introduction

Graphs are popular data structures that describe entities (or nodes) and their relationships (edges). Most real-world graphs are a mixture of hierarchical and non-hierarchical structures. Humans naturally use hierarchies to organize entity categories, for example, social networks, sentences in natural language, and evolutionary relationships in phylogenetics [20]. Typical hierarchical structures are denoted as a directed acyclic tree (e.g., an is-a relationship between abstractions such as “Elephant” is-a “Ungulate” and “Ungulate” is-a “Mammal”). As a motivating example, consider the articles published through ACM. Articles can cite each other (article-article link) and form a non-hierarchical structure. Each article is also associated with one or more ACM Computing Classification System (CCS) concepts, which consist of multiple trees of different depths. Thus, modeling the ACM graph necessitates handling both hierarchical and non-hierarchical structures.

Graph representation learning seeks to encode nodes as low-dimensional distributed vectors that can succinctly summarize the graph structure [11, 16, 17, 21, 23]. However, most graph representation learning approaches focus on modeling non-hierarchical structures by ignoring the hierarchical structures or considering the hierarchical (i.e., directed) links as an undirected form. One important characteristic of hierarchical structures is that the number of leaf nodes increases exponentially as the number of levels increases and can cause distortion issues when embedding such graphs [22]. Hyperbolic space has been proposed for representing latent hierarchical structures in graph-structured data [1, 4, 19, 20, 28, 29] as the volume grows exponentially with the radius and thus can naturally model the growth in leaf nodes.

**Figure 1:**
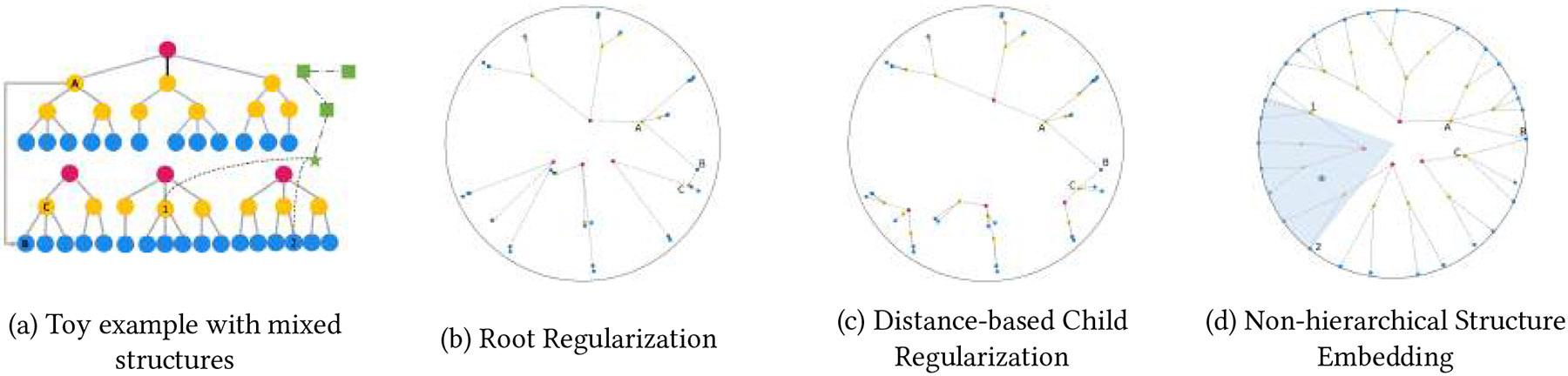
An example of the embedding results for the toy example (a) after applying each component. The circle nodes are from the hierarchical structure, and the star and square nodes are non-hierarchical structures. Some edges are not illustrated in (a) for simplicity. Note that non-hierarchical structures are not shown in (b) and (c). (d) shows the embedding results of using a hyperbolic entailment cone, and the shadowed area shows the region in the nodes in the non-hierarchical structure can reside.

Poincaré embedding model [19] is a popular embedding model in hyperbolic space. The learned node representations are defined within the n-dimensional Poincaré ball such that parallel points along two lines grow exponentially as the points get near the surface of the ball. The model implicitly learns the representations of the hierarchy such that root nodes generally lie at the origin while nodes at lower levels of the hierarchy will reside closer to the surface of the ball. Yet there are several limitations to existing models. First, they assume a hierarchical structure with a single root node and may not yield reasonable representations in the presence of multiple root nodes (e.g., multiple trees within CCS). Figure 1(a) shows an example of multiple root nodes that are depicted as a red circle. Second, when there is a poly-hierarchical structure (i.e., a child can have multiple parents from different trees), the implicit modeling of the hierarchy can result in representations where the child resides closer to the origin than the parent. Third, limited work considers graphs with mixed hierarchical and non-hierarchical structures [9]. Unfortunately, it relies on the product manifold of multiple hyperbolic, sphere, and Euclidean components, which leads to higher dimension sizes and increased computational costs. Last, recent works [5, 13, 14, 17] have focused on the semi-supervised or supervised setting, partly due to the advantages of graph neural networks. However, this often assumes labels and computational resources necessary to fine-tune are available.

To address the above limitations, we propose **HypMix**, an unsupervised **Hyp**erbolic representation learning model for graphs with **Mix**ed hierarchical and non-hierarchical structures. HypMix only relies on a single hyperbolic manifold. For graphs with hierarchical structures that contain multiple root nodes, we propose a regularization term to embed the root nodes close to the origin of the Poincaré ball. To tackle challenges related to poly-hierarchical structures, we propose two regularizations: (1) a distance-based restriction to embed parent nodes closer to the origin than their children and (2) using the hyperbolic entailment cone [7] to ensure two children reside in a similar Poincaré region. We also introduce the use of the hyperbolic entailment cone to the non-hierarchical structures to better embed these nodes in the Poincaré ball. We conduct extensive experiments across two evaluation tasks and three real-world datasets to demonstrate the effectiveness of HypMix over existing baselines. We also perform an ablation study to better understand the benefits of the three components of our model.

## 2 HypMix

HypMix adopts Poincaré embedding [19], which learns the representation of hierarchical structure into a hyperbolic space or an n-dimensional Poincaré ball. However, the basic Poincaré embedding model does not always learn the representation that preserves the hierarchical structure. For example, Poincaré embedding model cannot handle multiple root nodes which leads the root nodes to be placed in the outer part of the hyperbolic space than their child nodes. Also, because of the poly-hierarchical structures, some parent nodes are located further from the origin than their child nodes. Another limitation of Poincaré embedding is that it is a model only for hierarchical structures, which makes it challenging to learn the representation with non-hierarchical structures. To resolve these limitations, we use two regularizations to learn a better representation of the hierarchy structure and use hyperbolic entailment cone [7] also to learn the representation of non-hierarchical structures.

### 2.1 Root Regularization

One limitation of existing Poincaré-based models is the implicit design for a hierarchical structure with a limited number of roots (i.e., a small number of trees). However, some hierarchical taxonomies may have multiple categories or concepts that can be further separated into subcategories. For example, ACM CCS contains 13 root nodes (e.g., Networks, Theory of computation, Security and Privacy, etc.). Unfortunately, when the hierarchical structure encompasses multiple trees, the root embeddings of the tree may reside closer to the surface of the Poincaré ball. This restricts the embedding space to learn the hierarchical structure of subsequent children nodes and thus may result in suboptimal leaf embeddings.

To address this limitation for hierarchical structures with multiple root nodes, we propose a regularization term to encourage the root node to reside closer to the origin. In this manner, the subtree has sufficient space and more flexibility to better preserve deeper trees. Let the distance between two nodes, $u,v\in B^{d}$ where $B=\{x\in R^{d},\|x\|<1\}$ is the open d-dimensional unit ball and ‖⋅‖ denotes the Euclidean norm [19] be defined as:

(1) $$d(u,v)=arccosh(1+\frac{2{\left\|u-v\right\|}^{2}}{\left(1-{\left\|u\right\|}^{2})(1-{\left\|v\right\|}^{2}\right)})$$

Then, given a root node, $n_{root}$, we denote the distance to the origin, origin as $d(origin,n_{root})$ and impose the following condition:

(2) $$d(origin,n_{root})<\delta ,$$

where δ a user-specified parameter (shown in Figure 1(b)).

### 2.2 Child Regularizations

Another limitation of the Poincaré embedding model is that it only implicitly captures the hierarchical structure by modeling undirected edges. As such, it may not be able to distinguish which node is a child or parent and place child nodes closer to the origin than their parents. This is particularly difficult for a poly-hierarchical structure where a node may have parents from different trees. For example, ACM CCS is a poly-hierarchical ontology where concepts can belong to multiple categories. In this scenario, the ideal representation is the parent embedding, which resides closer to the origin than the child to reflect the hierarchical structure.

#### 2.1.1 Distance-based Child Regularization.

We first introduce a regularization term that restricts a parent from being further in distance from the origin than its child. Given two nodes, p and C where p is the parent node and C are the children nodes of p. We enforce HypMix to learn a representation using the distance between the two nodes, Eq. (1), that satisfies the following condition:

(3) $$d(origin,p)<d(origin,c_{i}),\;\forall c_{i}\in C(1\leq i\leq |C|).$$

Note that ∣C∣ denotes the number of children nodes of the parent node p. Figure 1(c) demonstrates the learned embedding after the child regularization is applied. We briefly note that the root regularization is not applied in this scenario. As shown in the figure, the child node resides further from the origin than its parent node and explicitly preserves the hierarchical structure where nodes at lower levels will be closer to the surface of the ball.

#### 2.2.2 Hyperbolic Entailment Cone Regularization.

The distance-based child regularization (Equation (3)) can help preserve the relationship between one parent and one child, yet two children of the same parent may not reside in a “similar” Poincaré region. Moreover, under the Poincaré embedding model, most points collapse on the border of the Poincaré ball. As such, we posit that a partial ordering where each subtree naturally defines the Poincaré region can further improve the learned embedding of the nodes within the tree. The idea is that a parent node will define a cone in the Poincaré space for which its children can reside and enable better differentiation of the node embeddings between multiple trees. Thus, if a child shares two parents, then it can only be nested in the intersection of the two cones defined by the parents. To achieve this, we leverage the hyperbolic entailment cone [7] to place the children nodes within the hyperbolic cones defined by the parent.

Hyperbolic entailment cones are inspired by the generalized idea of order embedding [25]. The idea is to use geodesically convex entailment cones to induce the partial ordering relation in the embedding space. The cones exhibit 4 intuitive properties that include axial symmetry, rotation invariance, continuous cone aperture functions, and transitivity of nested angular cones. Let $C_{x}$ denote the Poincaré entailment cone at apex $x\in B^{d}$ and be defined as:

(4) $$C_{x}=\left\{y\in B^{d}|\;{∠}_{xy}\leq \sin^{-1}(K\frac{1-{\left\|x\right\|}^{2}}{\left\|x\right\|})\right\},$$

where ${∠}_{xy}$ denotes the angle between the half-lines connecting x and y as well as the origin and x, and K∈R is a hyperparameter. Our entailment cone regularization then requires the following:

(5) $$c_{i}\in C_{p},\forall c_{i}\in C(1\leq i\leq |C|).$$

In other words, each child must belong to the angular cone defined by the parent. Figure 1(d) demonstrates the hyperbolic entailment cone in a hierarchical structure as illustrated by the circle nodes.

### 2.3 Non-hierarchical Structure Embedding

Across many real-world graphs, nodes may capture both hierarchical and non-hierarchical structures. The above regularizations (root, distance-based child, and hyperbolic entailment cone) can preserve the hierarchical structures, yet do not account for links to nodes that may not have a non-hierarchical structure. As a motivating example, consider articles published at the various ACM conferences. Each article can be tagged with multiple CCS categories (which exhibit a hierarchical structure), yet the articles themselves do not have a hierarchical structure. As such, the natural question is how to leverage the hierarchical structure to better embed the non-hierarchical nodes in the hyperbolic space.

Suppose we have two node types, $H=\{h_{1},h_{2},\ldots ,h_{n}\}$ and $V=\{v_{1},v_{2},\ldots ,v_{m}\}$, where there is a hierarchical relationship between the nodes in H while the nodes in V have non-hierarchical structure (i.e., can be linked to each other but not as a parent-child relationship), and there are also non-hierarchical links between H and V. Note that any nodes in V can have multiple relations with the nodes in H, and linked nodes in H can be located at any level in the tree. In this scenario, the hierarchical structure of H can serve as a guideline for learning the representation of the nodes in V. Our idea is that any node $v_{i}$ that is linked to a node in $h_{j}$ should then naturally reside in the same angular cone region defined by the node through the hyperbolic entailment cone:

(6) $$v_{i}\in C_{h_{j}},\;\forall v_{i}\in V\;and\;(v_{i},h_{j})$$

where $C_{h_{j}}$ is the entailment cone region defined using Eq. (4), and ($v_{i}$, $h_{j}$) denotes that the nodes $v_{i}$ and $h_{j}$ are linked. Thus, nodes in a non-hierarchical structure should also be embedded within the hyperbolic entailment cone of the associated hierarchical nodes. In this manner, hierarchical nodes that are indirectly linked together through a non-hierarchical node will reside in similar Poincaré space as the non-hierarchical must reside in the intersection. The blue area in Figure 1(d) is the region where the nodes in non-hierarchical structures can be located.

## 3 Experiment Settings

### 3.1 Evaluation Tasks

We use two evaluation tasks, systematic reviews (SRs) and node classification (NC). In health research, SRs are crucial for bridging the research-to-practice gap and serve as the basis for evidence-based practice [2, 3, 8]. Each article can be associated with multiple MeSH terms and the associated MeSH terms can be within the same MeSH hierarchy or tree (i.e., terms that are supported by a broader MeSH term) or can be in a different MeSH tree. We use the PGB benchmark dataset [18], and follow their experimental setting for evaluating the embedding model using three different SR datasets: Cohen [6], SWIFT-Review [12], and CLEF-TAR [15], where the goal is to predict whether the article passes the abstract screening process. Note that Cohen contains 15 SR topics, whereas the SWIFT-Review and CLEF-TAR both contain 3 SR topics each. For the SR task, we use 2 node types, *P*aper and *M*eSH terms, and 3 edge types, *P*-*P*, *P*-*M*, and *M*-*M*. Only the edge type, *M*-*M*, has a hierarchical structure, while the others are non-hierarchical.

For the NC task, we construct two datasets of real-world networks, DBLP^1^ and YELP^2^ with explicit hierarchical structure. For the DBLP dataset, we use the topic taxonomy from the ACM CCS codes, and the mapped information of authors and CCS codes are provided by Yang *et al*. [27]. We define a binary classification problem of whether the author has a link to one of the four selected CCS codes, “machine learning”, “artificial intelligence”, “information systems applications”, and “information retrieval” or not. The dataset has 2 node types, *A*uthor and CCS codes, and 3 edge types, *A*-*A*, *A*-C, and C-C where the only edge type C-C has the hierarchical structure, and others are non-hierarchical. For the YELP dataset, we follow the same setting as TAXOGAN [27], which generates the business network based on the customers’ reviews with the category of the business. We set it as a binary classification problem by setting two classes, stars > 3 and stars ≤ 3. The dataset has 2 node types, Business and Category, and 3 edge types, B-B, B-C, and C-C. From the edge types, C-C has a hierarchical structure, and other edge types are non-hierarchical. We randomly split the data into 3 train-test trials of 70%-30%, respectively.

### 3.2 Baseline Models

We benchmark HypMix with 4 baseline models. We also analyze three of the components that we propose. As HypMix is an unsupervised model, we compare it with an unsupervised network embedding models that use Euclidean space, LINE [24], and GraphSAGE (GS) [10], and hyperbolic space, Poincaré Embedding [19], and Hyperbolic Space Heterogeneous Network Embedding (HSHNE) [26]. We also compare three proposed components which are ${\text{HypMix}}_{R}$ (R), ${\text{HypMix}}_{Ch}$ (Ch), and ${\text{HypMix}}_{C}$ (C). ${\text{HypMix}}_{R}$ only applies the root regularization technique or Equation (2) and ${\text{HypMix}}_{Ch}$ only applies the child regularization technique, Equation (3). In addition to all the regularization techniques (root and child regularization), ${\text{HypMix}}_{C}$ uses the hyperbolic entailment cone to embed the hierarchical structure, Equation (5) but does not use it for non-hierarchical structures. We use a softmax layer to train the classifier. For the Euclidean space models, we use the dimension size 256 (d=256) for both SR and NC tasks. For the hyperbolic space models, we use d=50 for the SR task, and d=30 for the NC task due to the smaller size of the hierarchical structure. All the baselines are trained using a single g4dn AWS instance with NVIDIA T4 GPU.

## 4 Evaluations

The average AUC score on the three splits is reported in Table 1. Note that the first 21 datasets from the table are the SR tasks: Cohen, SWIFT-Review, and CLEF-TAR, respectively.

For the SR task, we observe that HypMix outperforms all other baselines from 0.002 to 0.037 by comparing with the second-best AUC score. This indicates the importance of effectively modeling both the hierarchical and non-hierarchical structures. Moreover, it demonstrates the effectiveness of HypMix in the SR task. Between the original Poincaré embedding model and HypMix, the results show that HypMix significantly outperforms the former and highlights the effectiveness of the components that we propose. It also shows that the original model cannot handle multiple trees and mixed node types. By comparing the results with LINE and GS (Euclidean space), HypMix outperforms both models, which illustrates the importance of using the hyperbolic space appropriately to embed hierarchical relations. Even if we use a larger dimension for both LINE and GS (d=256), they still fail to outperform HypMix. HSHNE outperforms Euclidean space models as HSHNE is a model to embed heterogeneous networks into a hyperbolic space which shows the effectiveness of the hyperbolic space.

For the NC task, we observe similar performance trends as the SR tasks in which ${\text{HypMix}}_{C}$ (C) and HypMix offer the best performance. We also observe that LINE and GS perform similarly to the Poincaré embedding results, potentially due to the smaller dimension size (d=30). This demonstrates the limitations of embedding hierarchical and non-hierarchical structures in Euclidean space. Since DBLP and YELP contain more tree-like hierarchical structures than the MeSH hierarchy, HSHNE and HypMix yield higher results compared to the SR task.

## 5 Conclusion

In this paper, we propose HypMix, an unsupervised hyperbolic representation learning for graphs with mixed hierarchical and non-hierarchical structures. We resolve the limitations of the Poincaré embedding model regarding handling multiple roots and poly-hierarchical structure. We propose root regularization to learn the representations of the root nodes to reside closer to the origin of the hyperbolic space. We also introduce two child regularizations so that the parent node is embedded closer to the origin than its child nodes and define the angular region, or entailment cone, for its children. Also, to learn the representation of the non-hierarchical structure, we adopt the hierarchical structure entailment cone to define the region of the non-hierarchical nodes. The extensive experiments on 21 real-world SR tasks and 2 real-world NC tasks show that the HypMix outperforms existing unsupervised graph representation learning models.

## Figures and Tables

**Table 1:** AUC performance for the SR and NC tasks. The best score is bolded and the second highest is underlined. The SR topics follow the same order as the PGB results [18].

Dataset	LINE	GS	Poincaré	HSHNE	R	Ch	C	HypMix
ACE	0.544	0.546	0.524	0.586	0.534	0.532	0.556	**0.589**
ADHD	0.533	0.537	0.522	0.540	0.523	0.533	0.539	**0.552**
AH	0.541	0.543	0.518	0.547	0.514	0.534	0.547	**0.567**
AAP	0.547	0.547	0.522	0.555	0.523	0.534	0.552	**0.561**
BB	0.564	0.568	0.554	0.584	0.551	0.555	0.579	**0.59**
CCB	0.566	0.57	0.549	0.597	0.555	0.559	0.581	**0.599**
Estrogens	0.534	0.536	0.53	0.543	0.529	0.534	0.539	**0.548**
NSAIDS	0.549	0.553	0.536	0.578	0.535	0.54	0.568	**0.588**
Opioids	0.557	0.561	0.544	0.604	0.539	0.546	0.583	**0.606**
OH	0.509	0.508	0.502	0.530	0.502	0.504	0.51	**0.535**
PPI	0.558	0.56	0.523	0.586	0.527	0.533	0.585	**0.61**
SKM	0.562	0.565	0.534	0.582	0.532	0.542	0.581	**0.612**
Statins	0.549	0.551	0.534	0.573	0.543	0.542	0.558	**0.577**
Triptans	0.551	0.554	0.53	0.592	0.534	0.544	0.565	**0.596**
UTI	0.556	0.559	0.537	0.572	0.542	0.543	0.569	**0.609**
TG	0.579	0.584	0.566	0.643	0.579	0.577	0.632	**0.645**
PFOS-PFOA	0.582	0.584	0.572	0.630	0.581	0.573	0.622	**0.641**
BPA	0.546	0.545	0.518	0.561	0.524	0.523	0.552	**0.57**
CD012661	0.547	0.552	0.532	0.590	0.54	0.538	0.576	**0.598**
CD008803	0.552	0.561	0.544	0.590	0.554	0.552	0.579	**0.604**
CD005139	0.573	0.583	0.556	0.605	0.566	0.561	0.596	**0.627**
DBLP	0.581	0.583	0.578	0.643	0.576	0.579	0.632	**0.657**
YELP	0.555	0.556	0.545	0.598	0.545	0.546	0.589	**0.602**

## CCS Concepts

- Information systems → Hierarchical data models; Network data models.

## Footnotes

1
https://dblp.uni-trier.de/xml/

2
https://www.yelp.com/dataset
